# Comparative Analysis of RNA Seq and PCR Array Data Implicates BDNF as a Potential Therapeutic Target for Alzheimer's Disease

**DOI:** 10.1002/alz70855_101455

**Published:** 2025-12-23

**Authors:** James P Owens, Frank J Castora

**Affiliations:** ^1^ Old Dominion University, Norfolk, VA, USA; ^2^ Macon and Joan Brock Virginia Health Sciences Eastern Virginia Medical School at Old Dominion University, Norfolk, VA, USA

## Abstract

**Background:**

A mutation in mitochondrial DNA (mtDNA) that is associated with Alzheimer's disease (AD) has been reported by us. We compared human brain RNA expression data from PCR array and bulk RNA seq analyses to understand effects of the T9861C mutation on gene expression and to help identify potential therapeutic targets for AD and AD+ patients.

**Method:**

Qiagen Ingenuity Pathway Analysis (IPA) software was used to compare each gene as three expression log ratios: AD/control brains, AD+/control brains, and AD+/AD brains. The two analyses were compared. Regulator effects were grown to connect with other molecules, and the downstream and upstream effects of activating and inhibiting molecules were observed using the molecule activity predictor (MAP). We created a pathway using regulators and molecules directly related to AD and used MAP to identify potential therapeutic targets.

**Result:**

Our analysis identified the top canonical pathways, the most expressed regulators, the top diseases and functions affected by the expression of the genes, and the downstream and upstream effects of the changing expression of genes in our datasets. The RNA bulk sequencing provided more data, resulting in IPA returning more canonical pathways, regulators, regulator effects, diseases and functions related to the genes in the datasets. These results were less statistically significant than those returned by the PCR array analysis. The AD/Control ratio was the most statistically significant for PCR array analysis while AD+/Control was the most statistically significant for RNA bulk sequencing analysis.

**Conclusion:**

The difference in the amount of data and statistical significance provided by the RNA bulk sequencing and the PCR array analyses provides evidence that the focused number of genes in the PCR array analysis results in greater statistical significance while sacrificing breadth. Through manipulating the expression of regulators, molecules, and diseases for the two datasets, we identified activating **brain derived neurotrophic factor (BDNF)** as the most effective therapeutic approach for inhibiting Alzheimer's disease. BDNF will be added to a mathematical model for AD that we are constructing to evaluate and optimize the therapeutic potential of activating BDNF to significantly inhibit AD in AD or AD+ patients.

